# The structure of *Leptospira interrogans* GAPDH sheds light into an immunoevasion factor that can target the anaphylatoxin C5a of innate immunity

**DOI:** 10.3389/fimmu.2023.1190943

**Published:** 2023-06-20

**Authors:** Sergio Navas-Yuste, Karla de la Paz, Javier Querol-García, Sara Gómez-Quevedo, Santiago Rodríguez de Córdoba, Francisco J. Fernández, M. Cristina Vega

**Affiliations:** ^1^Centro de Investigaciones Biológicas Margarita Salas, Consejo Superior de Investigaciones Científicas (CSIC), Madrid, Spain; ^2^Abvance Biotech Srl, Madrid, Spain; ^3^Universidad Europea, Madrid, Spain; ^4^Centro de Investigación Biomedica en Red sobre Enfermedades Raras (CIBERER), Madrid, Spain

**Keywords:** structural biology, innate immunity, complement system, C5a anaphylatoxin, GAPDH – glyceraldehyde 3-phospate dehydrogenase, leptospirosis, moonlighting proteins

## Abstract

Leptospirosis is a neglected worldwide zoonosis involving farm animals and domestic pets caused by the Gram-negative spirochete *Leptospira interrogans*. This bacterium deploys a variety of immune evasive mechanisms, some of them targeted at the complement system of the host’s innate immunity. In this work, we have solved the X-ray crystallographic structure of *L. interrogans* glyceraldehyde-3-phosphate dehydrogenase (GAPDH) to 2.37-Å resolution, a glycolytic enzyme that has been shown to exhibit moonlighting functions that potentiate infectivity and immune evasion in various pathogenic organisms. Besides, we have characterized the enzyme’s kinetic parameters toward the cognate substrates and have proven that the two natural products anacardic acid and curcumin are able to inhibit *L. interrogans* GAPDH at micromolar concentration through a noncompetitive inhibition modality. Furthermore, we have established that *L. interrogans* GAPDH can interact with the anaphylatoxin C5a of human innate immunity in vitro using bio-layer interferometry and a short-range cross-linking reagent that tethers free thiol groups in protein complexes. To shed light into the interaction between *L. interrogans* GAPDH and C5a, we have also carried out cross-link guided protein-protein docking. These results suggest that *L. interrogans* could be placed in the growing list of bacterial pathogens that exploit glycolytic enzymes as extracellular immune evasive factors. Analysis of the docking results indicates a low affinity interaction that is consistent with previous evidence, including known binding modes of other α-helical proteins with GAPDH. These findings allow us to propose *L. interrogans* GAPDH as a potential immune evasive factor targeting the complement system.

## 1 Introduction

The complement system is a central part of the innate immune defense against pathogens (1). It comprises about forty soluble and membrane-associated proteins, which survey the blood and interstitial fluids for pathogens, immune complexes, and apoptotic cell debris. Those stimuli can activate the complement system very swiftly through three main activation pathways: the alternative (AP), classical (CP), and lectin (LP) pathways. Normal complement activation on surfaces involves a self-amplification cascade where the so-called C3 convertases proteolytically cleave C3, the most abundant complement factor, to yield C3b, which remains attached to the activating surface, and C3a. Surface-attached C3b can assemble C3-convertase enzyme complexes, propagating C3b deposition in a process known as opsonization. C3b can be quickly cleaved by factor I into iC3b (2). On densely opsonized surfaces like those of pathogens and other foreign or damaged surfaces, C3b-containing enzyme complexes can cleave C5 into C5b and soluble C5a, a 74-amino-acid anaphylatoxin (3). The former remains bound to surfaces and nucleate the assembly of the so-called membrane attack complex (MAC) (4), which can lyse targeted cells directly through osmotic shock in a process known as terminal pathway. Like C3a, C5a is a soluble factor that diffuses away from the site of activation and acts as one of the most powerful chemoattractants of innate immunity. Once liganded to its cognate receptor (C5aR1/CD88), C5a stimulates proinflammatory responses like chemotaxis and vascular permeability, which result in the recruitment of inflammatory neutrophils and macrophages to the sites of activation (5). On self-cells, however, complement activation is strongly suppressed by self-protective fluid-phase regulators such as factor H and C4b binding protein (C4BP), both involved in the inactivation of iC3b on opsonized cell surfaces, and membrane regulators such as MCP and DAF, which disassemble C3 convertases to prevent further deposition of C3b (6).

Leptospirosis is a widespread zoonotic disease caused by the highly motile Gram-negative spirochete *Leptospira* (7, 8). *Leptospira* colonizes a range of hosts including humans, domestic and farm animals, and some wild animal species such as mice, rats, and bats, which typically serve as reservoirs of infection (9). In humans, leptospirosis typically presents with mild fever and flu-like symptoms, but in its more severe forms it can lead to fatal multi-organ failure. Leptospirosis causes about 1 million severe cases in humans every year with 60,000 fatalities (10). In cattle and swine, leptospirosis causes veterinary and economic damage through reproductive failure, abortion, still-births, fetal mummification, weak calves/piglets, and agalactia (8, 11). Its prevalence has surged in recent years due to global warming, intensive farming, and other geographic and socioeconomic factors (12). Globally, leptospirosis represents an increasing public and veterinary health threat, as evidenced by growing incidence rates and multiple outbreaks around the world, compounded by frequent misdiagnosis.

Pathogenic *Leptospira* produces the activation of all three complement activation pathways and, not surprisingly, it has evolved sophisticated immune evasion mechanisms to escape it (13). The deployment of *Leptospira*’s complement-targeting molecular weaponry accelerates the decay of the three complement activation pathways and inhibits the terminal pathway, thereby promoting the pathogen’s dissemination and infection. Examples of immunoevasion strategies deployed by *Leptospira* include: 1) acquisition *via* surface evasion molecules of host’s soluble complement regulators like factor H and C4BP (molecular mimicry) (14); 2) terminal pathway inhibition either through the direct interaction of surface pathogenic proteins with C9 or through the indirect interaction with vitronectin, an inhibitor of C5b7 complex formation and C9 polymerization; 3) plasminogen binding to and cleavage of C3b, C4b, and C5 (mixed molecular mimicry/proteolytic cleavage) (15); and 4) direct proteolytic degradation of complement proteins C2, C3, C4, and factor B. Several *Leptospira* virulence factors comprising extracellular enzymes and cell-surface proteins have been demonstrated to play key roles in host-cell adherence and immunoevasion. To date, two *Leptospira* proteins displaying moonlighting functions have been found: elongation factor-thermal unstable (EF-Tu), shown to interact with host extracellular membrane (ECM) molecules, plasminogen, and factor H, and the glycolytic enzyme α-enolase, described to interact with plasminogen, factor H, and C4BP.

We and others have proposed that the ubiquitous glycolytic enzyme glyceraldehyde-3-phosphate dehydrogenase (GAPDH; E.C. 1.2.1.12) from pathogenic bacteria may double as an innate immune evasive factor when it is found in the extracellular environment (16). To perform these moonlighting functions, GAPDH must be relocated to the extracellular space by cell lysis (e.g., *via* streptococcal lysins) (16), secretion (e.g., type-3 secretion systems in enteropathogenic *Escherichia coli*) (17), or outer membrane shedding (e.g., *Francisella tularensis*, *Mycobacterium tuberculosis*, *Staphylococcus aureus*, *Atopobium vaginae*, and *Leptospira interrogans*). Some of the infectivity enhancing functions attributed to moonlighting GAPDH are mostly targeted at the innate immunity and, specifically, the complement system. Examples of these mechanisms include sequestering nascent C5a as it is being generated by C5 cleavage, a mechanism described for Gram-positive bacteria such as *Streptococcus pneumoniae*, *S. pyogenes*, *A. vaginae*, and *Clostridium perfringens* (16, 18, 19); binding complement factors like C3 and C1q (20, 21); and increasing pathogen dissemination by binding to ECM components as plasminogen to help to degrade tissue barriers, basement membranes, and fibrin clots (13, 22).

In this work, we set out to characterize the structure and function of GAPDH from the Gram-negative spirochete *L. interrogans* and investigate whether it could operate as a virulence factor by binding to C5a. We have characterized *Li*GAPDH’s enzymatic activity and inhibition by curcumin and anacardic acid, two natural products, and we have solved its crystal structure at 2.37-Å resolution complete with its NAD^+^ cofactor. Furthermore, we have shown by bio-layer interferometry and controlled cross-linking experiments that *Li*GAPDH can bind C5a, a property shared by GAPDH enzymes from other pathogenic bacteria that might contribute to immune evasion in the mammalian host. To shed light into the C5a recognition mechanism, we have performed cross-link guided protein-protein docking.

## 2 Materials and methods

### 2.1 Cloning, expression, and purification of *Li*GAPDH

The gene encoding full-length *Li*GAPDH (UniProt Accession No. Q72QM3_LEPIC) was amplified by PCR from *L. interrogans* serovar Copenhageni strain Fiocruz L1-130 genomic DNA (ATCC) and cloned into the pETM-11 expression vector by restriction-ligation after digesting the PCR fragment with BsaI-XhoI and the pETM-11 expression vector with NcoI-XhoI, conferring an N-terminal hexahistidine tag and a tobacco etch virus cleavage (TEV) site in frame with the *Li*GAPDH gene. The expression plasmid was verified by sequencing the entire ORF. For protein expression, the *Li*GAPDH construct was transformed into Rosetta(DE3) chemically competent cells. An overnight starter culture was used to inoculate a 2.5-L expression culture at 37 °C. The culture was allowed to grow at 37 °C in Luria-Bertani medium supplemented with 50 µg/ml kanamycin and 34 μg/ml chloramphenicol to an absorbance of 0.6 at 590 nm and then induced with 1 m*M* isopropyl β-D-thiogalactopyranoside (IPTG) for 20 h. Cell pellet was lysed by sonication in IMAC-A buffer (50 m*M* Tris-HCl (pH 8.0), 500 m*M* NaCl, 20 m*M* imidazole) supplemented with 1 mM phenylmethylsulfonyl fluoride (PMSF) and one tablet of EDTA-free protease inhibitor cocktail. Supernatant was collected upon centrifugation for 20 min at 4 °C. The sample was clarified further by filtration through a 0.22 µm membrane and loaded on a HisTrap column (Cytiva) pre-equilibrated in IMAC-A buffer and eluted in IMAC-A buffer with 250 m*M* imidazole. Peak fractions were analyzed by SDS-PAGE and fractions containing *Li*GAPDH were pooled and dialyzed against a buffer containing 10 m*M* HEPES-NaOH (pH 7.4), 150 m*M* NaCl, 3.4 m*M* EDTA. Then, *Li*GAPDH was subjected to size exclusion chromatography on a HiLoad 16/60 Superdex 200 pg (Cytiva) pre-equilibrated in the same buffer. Comparison of the elution volume of *Li*GAPDH with a calibration curve constructed using high and low molecular weight calibration kits (Cytiva) revealed that the quaternary structure of *Li*GAPDH corresponds to a tetrameric oligomeric state. Finally, *Li*GAPDH was concentrated to 10 mg/ml, dispensed in 50-µl aliquots, snap-frozen in liquid nitrogen and stored at −80 °C until use. The yield was ~2 mg *Li*GAPDH/l of culture, showing a >95% purity on a Coomassie brilliant blue-stained SDS-PAGE gel.

### 2.2 Enzyme kinetics

The *Li*GAPDH enzyme activity was followed spectrophotometrically by the change in absorbance at 340 nm due to NADH formation (ϵ = 6220 M^−1^ cm^−1^), adapted from a previously described method (23). Temperature controlled assays were performed in an Eppendorf BioSpectrometer spectrophotometer at 25 °C. One unit of enzyme activity was defined as the amount of GAPDH that converts 1 µmol/min of NAD^+^ to NADH at 25 °C. A standard assay was carried out in a final volume of 0.15 ml using 40 m*M* Tris-HCl (pH 8.5), 2 m*M* EDTA, 10 n*M Li*GAPDH, and indicated concentrations of the different substrates: nicotinamide adenine dinucleotide (NAD^+^), glyceraldehyde 3-phosphate (G3P), and inorganic phosphate (P_i_). NAD^+^ concentration was varied between 0.02 and 1.62 m*M* while keeping fixed G3P at 2 m*M* and P_i_ at 5 m*M*; G3P concentration between 0.14 and 11.7 m*M* at 2 m*M* NAD^+^ and 5 m*M* Pi; and P_i_ concentration between 0.38 and 31.5 m*M* at 2 m*M* NAD^+^ and 2 m*M* G3P. The reaction was initiated by adding 0.56 μg of enzyme. Michaelis-Menten parameters were obtained by non-linear regression fitting of the kinetic data using SigmaPlot 14.5 (Systat Software Inc.).

### 2.3 Inhibition by curcumin and anacardic acid

Inhibition assays were performed with two natural compounds, anacardic acid and curcumin. Four inhibitor concentrations each were tested for anacardic acid (10, 24, 64, and 160 μ*M*) and curcumin (10, 25, 62.5, and 150 μ*M*). To ascertain the inhibition modality with respect to G3P and NAD^+^, initial velocity measurements at each inhibitor concentration were carried out varying G3P concentration (0.06-1.56 m*M* G3P for both inhibitors) while maintaining a saturating concentration of 2 m*M* NAD^+^; or varying NAD^+^ concentration (0.02-1.1 m*M* NAD^+^ for both inhibitors) while maintaining a saturating concentration of 2 m*M* G3P. In either case, potassium phosphate was kept at a saturating concentration of 5 m*M*. The software SigmaPlot 14.5 (Systat Software Inc.) was used to analyze the data.

### 2.4 Crystallization and X-ray diffraction data collection

To find the optimal crystallization conditions, we performed extensive scans of crystallization conditions from commercial screenings by Hampton Research and Molecular Dimensions (Crystal Screening 1 and 2, Salt Screening 1 and 2, JSCG+ 1 and 2). The best results were obtained with Bis-Tris buffer at different concentrations of PEG 3350 as precipitant. The crystals presented a form of elongated prisms with a dimension of 150-300 μm in their longest axis. For freezing, 20% (v/v) glycerol was used as cryoprotectant. Crystals were mounted on Micromount loops (MiTeGen) and frozen in liquid nitrogen. The crystals were diffracted at the BL13-XALOC beamline of the ALBA synchrotron (Barcelona) (24). The maximum observable resolution was 2.37 Å with unit cell dimensions of *a* = 79.8 Å, *b* = 82.0 Å, *c* = 123.2 Å, *α* = 94.0°, *β* = 95.1°, and *γ* = 112.5° and *P*1 space group. The data was processed with *XDS* (25) and scaled and merged with *Aimless* (26).

### 2.5 Structure determination

The crystallographic structure of *Li*GAPDH was solved by molecular replacement using *PHASER* (27) in the *PHENIX* suite (28) with the structure of *Av*GAPDH as a model (PDB ID 5LD5; http://doi.org/10.2210/pdb5LD5/pdb) (18). The crystal structure contained two tetramers in the asymmetric unit. The difference map (*F*_o_-*F*_c_) showed a clear position and conformation for an NAD^+^ cofactor in the active site of all the monomers from the two tetramers. Refinement cycles with phenix.refine (29) of the *PHENIX* suite were interspersed with cycles of manual construction (placing first NAD^+^ and then solvent molecules) and validation cycles with *Coot* (30). Non-crystallographic symmetry was applied as a constraint on the main-chain dihedral angles during the initial refinement, but they were removed during the later stages of refinement. At the end of the refinement, the *Li*GAPDH model obtained a *R*_work_/*R*_free_ of 0.19/0.23 with an r.m.s.d. of 0.011 Å and 1.31° for distance and bond angles, respectively. The final model consists of 2674 amino acid residues, eight NAD^+^ molecules, 380 water molecules, 4 phosphate anions, and 33 glycerol molecules from the cryoprotectant solution. The crystallographic refinement statistics are summarized in **Table 1**.

**Table 1:** Data collection and refinement statistics (molecular replacement).

	*Li*GAPDH
Data collection	
Space group	*P*1
Cell dimensions	
*a*, *b*, *c* (Å)	79.80, 82.04, 123.19
α, β, γ (°)	94.02, 95.15, 112.52
Resolution (Å)	45.5–2.37 (2.43–2.37)*
No. total reflections	401,101 (27,157)
No. unique reflections	109,875 (7248)
Mean *I/*σ*I*	8.10 (0.91)
*R*_merge_	0.1077 (1.356)
*R*_meas_	0.1262 (1.581)
CC1/2	0.996 (0.401)
Completeness (%)	94.76 (87.31)
Redundancy	3.6 (3.7)
Refinement	
No. reflections	109,875 (7248)
No. reflections in test set	2008 (131)
*R*_work_/*R*_free_	0.1923/0.2322
No. residues	2674
No. atoms	21,539
Protein	20,540
Ligand/ion	619
Water	380
*B*-factors (Å^2^)	61.12
Protein	61.32
Ligand/ion	57.95
Water	55.49
R.m.s. deviations	
Bond lengths (Å)	0.011
Bond angles (°)	1.31
Ramachandran plot	
Favored (%)	97.84
Allowed (%)	1.82
Outliers (%)	0.34
Rotamer outliers (%)	1.00
Clashscore	6.00

The structure was determined from a single crystal.

* Values in parentheses are for the highest-resolution shell.

The coordinate and structure factors files have been deposited with the Protein Data Bank (PDB) with PDB ID 8OHA (http://doi.org/10.2210/pdb8OHA/pdb).

### 2.6 Small angle X-ray scattering

SAXS experiments were carried out at the B21 beamline (31) from the Diamond Light Source synchrotron (DLS, UK). To improve sample purity and monodispersity, we collected SAXS data using continuous flow in HPLC-SAXS mode (620 images/3 s) at 9 °C. Sample *Li*GAPDH at 7 mg/ml in 10 m*M* HEPES-NaOH (pH 7.4), 150 m*M* NaCl, 3.4 m*M* EDTA, 2 m*M* TCEP, 3% (v/v) glycerol were injected on a Shodex KW-403 size-exclusion column (theoretical separation range 10-700 kDa, 4.6-ml column volume), previously equilibrated in the same buffer. Individual 2D data images were radially averaged to produce 1D diffraction profiles *I*(*q*) *vs. q* without subtracting buffer. For the final data reduction process, statistical checks were performed to rule out images affected by radiation damage or systematic scaling errors (32). The data were averaged, buffer subtracted, and combined to produce the final SAXS profile covering the transfer momentum range 0.0026 to 0.3400 Å^-1^. The *ATSAS* 3.0 software package (33) was used to extract structural information and perform an *ab initio* shape restoration of *Li*GAPDH. Firstly, the number of Shannon channels and the maximum usable *q* were estimated with *SHANUM* (34). Next, the direct diffraction extrapolated to zero angle *I(0*) and the *R*_g_ were evaluated using the Guinier approximation (35) and the pairwise distance distribution function in real space (*P*(*r*) *vs. r*) computed with *GNOM* (36). From the *P*(*r*) profile it was possible to evaluate the maximum dimension (*D*_max_) of the particle. Additionally, two different concentration-independent methods were used to estimate the molecular mass of *Li*GAPDH: the correlation volume (*V*_C_) and the empirical Porod volume (*V*_P_) correction (37), implemented in the *ATSAS* toolsets called *DATMOW*, *DATVC*, and *DATPOROD*. With *DATCLASS*, the shape of *Li*GAPDH derived from the SAXS data was classified as compact and potentially unique (38). *Ab initio* shape restoration was performed using a dummy bead model of 50 independent runs with *DAMMIF* (39), which were superimposed, averaged, and clustered with *DAMAVER* (40), and further refined with *DAMMIN* (41) to create the final *ab initio* shape. The most representative cluster contained >90% of all bead models with a normalized spatial discrepancy (NSD) threshold of 0.55 (42). The resolution was estimated from the Fourier shell correlation (FSC) at FSC = 0.5 (43). The fit of the crystal structure of *Li*GAPDH to the SAXS data was evaluated using *CRYSOL* (44).

### 2.7 Bio-layer interferometry

Bio-layer interferometry (BLI) studies were performed on a BLItz instrument (ForteBio) at 25 °C with shaking at 2200 rpm. BLI assay buffer consisted of 10 m*M* HEPES-NaOH (pH 7.4), 50 m*M* NaCl, 0.34 m*M* EDTA, 0.02% (v/v) polysorbate 20 (P20), which was 0.22-µm filtered. Before use, (anti-biotin) streptavidin (SA) biosensors (ForteBio 18-5019) were hydrated in BLI assay buffer for 10 min. All samples for BLI measurements were prepared in 4.5 µl. The BLI assay was as follows: baseline (30 s) (Equilibration), loading (300 s) (BLI assay buffer for nonspecific binding or biotinylated-C5a (34 µg/ml) (Abvance Biotech ABVC5ARBIO1), stabilization (300 s) (BLI assay buffer), baseline (30 s) (Equilibration), association (300 s) (230 µM *Li*GAPDH), and dissociation (300 s) (BLI assay buffer). Loading of biotinylated-C5a for 300 s onto SA sensor tips resulted in a wavelength shift signal of ~2.75 nm. Loading of *Li*GAPDH for 300 s onto either the mock SA sensor tips or the biotinylated-C5a SA sensor tips resulted in wavelength shift signals of 0.5 and 4.5 nm, respectively.

### 2.8 Cross-linking

Specific cross-linking assays were performed with bis(maleimido)ethane (BMOE; Pierce 22322), a 7-atom, 8.0-Å short spacer arm cross-linking reagent that generates non-cleavable cross-links between sulfhydryl groups that are in proximity (< 8 Å apart). Cross-linking reactions were carried out by mixing 10 µg *Li*GAPDH with 0.5, 1.0, 2.0, 4.0, or 5.0 µg human recombinant C5a (ABVC5A, Abvance Biotech) in assay buffer (10 m*M* HEPES-NaOH (pH 7.4), 150 m*M* NaCl, 3.4 m*M* EDTA) containing 0.3 m*M* BMOE, and incubating the reaction mixtures for 2 h at 4 °C. The C5a:*Li*GAPDH molar ratio for these assays was 0.27, 0.53, 1.07, 2.13, or 2.67; and the BMOE:*Li*GAPDH molar ratio was 1.44. To control for nonspecific cross-linking, we treated identical amounts of *Li*GAPDH (10 µg) and C5a (5 µg) with 0.3 m*M* BMOE and without BMOE. We followed the appearance of cross-linked products by 12% SDS-PAGE gel electrophoresis and Coomassie-Brilliant Blue (CBB) staining. A second SDS-PAGE gel was run with one-fourth of the cross-linking reactions under otherwise identical conditions and transferred onto a nitrocellulose membrane (1 h at 80 V) for Western blotting. The membrane was incubated 1 h at RT with blocking solution (5% (w/v) BSA in TBST), probed with an anti-C5a primary antibody (1:6000, 1 h at 37 °C) and a Goat anti-Rabbit HRP secondary antibody (1:2000, 30 min at 37 °C), developed with luminol and water peroxide, and imaged on a ChemiDoc Imaging System (Bio-Rad). Afterwards, the same blot was treated with Restore Stripping buffer (ThermoFisher Scientific 21059) for 30 min at 37 °C, reblocked, reprobed with an anti-His HRP antibody (1:3000, 1 h at RT), developed with luminol and water peroxide, and imaged.

### 2.9 Cross-link guided docking protocol

We applied a cross-link guided protein-protein docking protocol to predict *Li*GAPDH-C5a complexes using the standard *ROSETTA* docking protocol (45), with modifications. Receptor (*Li*GAPDH) and ligand (C5a) were first relaxed and then subjected to cross-link guided docking using the known length of the BMOE cross-link as restraint. The protocol filters and ranks the protein-protein docking poses by the sequential application of Xwalk (46) to simulate cross-links on protein surfaces, FreeSASA (47) and *PISA* (48) to calculate the size of predicted binding interfaces, and an energy-based clustering approach implemented in *ROSETTA* (49).

### 2.10 Electrostatic potential calculations

Electrostatic potential surfaces of *Li*GAPDH and C5a were calculated with the APBS (Adaptive Poisson-Boltzmann Solver) (50) software as a plugin in PyMOL (51) using default parameters.

## 3 Results

### 3.1 Crystallographic structure of *Li*GAPDH

**Figure 1:**
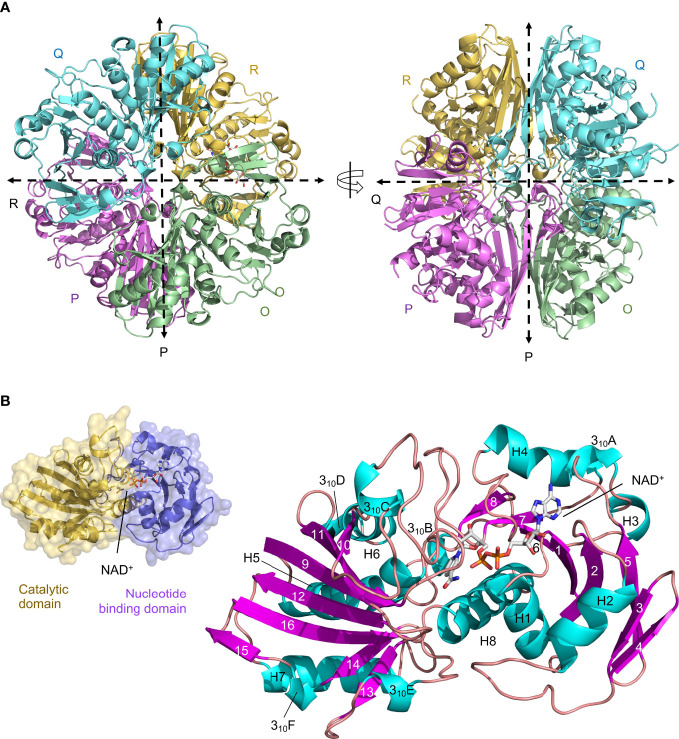
Crystal structure of *Li*GAPDH. **(A)** Overall tetrameric structure of *Li*GAPDH in cartoon representation with chain colors (O in green, P in violet, Q in cyan, and R in yellow). Crossing dashed lines indicate the directions of the two molecular symmetry axes on the plane of the figure. The NAD^+^ cofactor is shown in sticks and CPK colors. Two views of the *Li*GAPDH tetramer are shown down the Q-axis or the R-axis, which are perpendicular to the plane of the figure. **(B)** Cartoon representation of the *Li*GAPDH subunit structure color coded according to secondary structure: helices in cyan, β-strands in violet, and irregular segments and loops in salmon. Secondary structural elements and the NAD^+^ are annotated. In the inset (top left corner) we show the same monomer in molecular surface representation, color according to the domain: the *N*-terminal nucleotide-binding domain is in violet and the *C*-terminal catalytic domain in gold.

We have determined the first crystal structure of *Li*GAPDH at 2.37-Å resolution (**Figure 1**). The crystal structure corresponds to the holoenzyme with an NAD^+^ molecule tightly bound into the active site (**Figure 1**). We solved the structure by molecular replacement using *Atopobium vaginae* GAPDH as a model (*Av*GAPDH; PDB ID 5LD5; http://doi.org/10.2210/pdb5LD5/pdb) (18). There are two independent tetramers in the asymmetric unit that are nearly identical, with an r.m.s.d. of 0.30 Å. This remarkable similarity is mirrored by the structure of the individual subunits, which can be superimposed with an r.m.s.d. of 0.26 Å on average. Crystallographic data processing and refinement and validation statistics are reported in **Table 1**.

The quaternary structure of *Li*GAPDH consists in a homotetramer with O, P, Q, and R subunits related by a 222/*D*2 molecular symmetry, which gives rise to three non-equivalent interfaces related by three mutually perpendicular axes referred to as P, Q, and R (**Figure 1A**). The monomers are composed of two domains: an *N*-terminal domain that contains the NAD^+^ binding pocket (residues 1-152), and a catalytic *C*-terminal domain spanning residues 153-335 (**Figure 1B**). The *N*-terminal domain adopts an α/β/α Rossmann fold characterized by the classic α/β nucleotide binding pocket, which typically contains a central 7-stranded β-sheet and a tightly bound NAD^+^ cofactor occupying the active site. In *Li*GAPDH there are 8 β-strands (β1 to β8) because the canonical seventh β-strand is split into two smaller β-strands (β7 and β8) by a small irregular segment of extended conformation. Small helices are inserted between consecutive β-strands in this domain, which is further stabilized by packing against the *C*-terminal H8 helix. The *C*-terminal domain contains an 8-stranded β-sheet (β9 to β16), with helices inserted between β9-β10 (3_10_C), β10-β11 (H6 and 3_10_D), β12-β13 (H7), and β13-β14 (3_10_E). Helix H8 is the last secondary structural motif of *Li*GAPDH.

The main interfaces through which each *Li*GAPDH monomer interacts with its two neighboring chains within the tetramer are not equivalent and have different surface areas (**Figure 1A** and **Supplementary Figures 1**, **2**). First, the interface between the O-P subunits is the most extensive, with an average surface area of 1901 Å^2^ (**Supplementary Figure 1**). Nine H-bonds and 19 salt bridges stabilize the O-P interface. Second, the O-R interface, with an average surface area of 1412 Å^2^, has up to 13 H-bonds (**Supplementary Figure 2A**). Finally, the smallest intersubunit interface lies between the O-Q subunits, with an average surface area of 492 Å^2^, 10 H-bonds, and 2 salt bridges (**Supplementary Figure 2B**).

Another key structural feature of *Li*GAPDH is the *S* loop, an extended and irregular segment comprising residues Ala180-Ile207 that inserts itself between the NAD^+^-binding site and the adjacent subunit (**Figure 1B** and **Supplementary Figure 2**). The *S* loop contains residues lying between two of the catalytic triad residues His179 and Arg234 that are important for catalysis, cofactor-binding, and dimerization.

### 3.2 Solution SAXS shape of *Li*GAPDH

**Figure 2:**
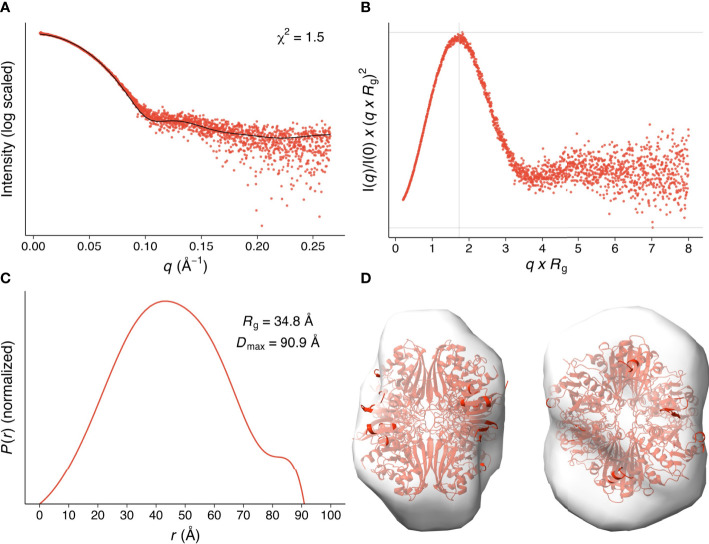
SAXS hydrodynamic properties and shape restoration for *Li*GAPDH. **(A)** 1D diffraction intensity of *Li*GAPDH plotted as a function of the diffraction momentum transfer *q*. Experimental data shown as a scatter plot (red circles). The black line corresponds to the average of the theoretical scattering profile of the two *Li*GAPDH tetramers in the asymmetric unit (*χ*^2 ^= 1.5). **(B)** Dimensionless Kratky representation showing the degree of protein folding in solution. The experimental pattern is shown as a scatter plot (red circles). The cross (gray lines) marks the so-called Guinier-Kratky point (1.732, 1.1), i.e., where the position of the main peak for globular proteins would be located. **(C)** Pair distance distribution function *P*(*r*) plotted as a function of *r*. The experimental pattern is shown as a solid line (red color). The value of *D*_max_ is the largest non-negative value that the distribution function supports. **(D)** Cartoon representation of *Li*GAPDH (in red) fitted inside the *ab initio* shape calculated with *DAMMIF*. Two orientations 90° apart are shown.

We analyzed the size and other hydrodynamic properties of *Li*GAPDH by solution small-angle X-ray scattering (SAXS) at the B21 beamline of the Diamond Light Synchrotron (DLS) (31). SAXS parameters are reported in **Table 2** and **Supplementary Table 1**. Results indicated that *Li*GAPDH is a fairly spherical homotetramer with a well-folded structure (**Figures 2A, B**), with a radius of gyration *R*_g_ of 34.8 Å and a maximum dimension *D*_max_ of 90.9 Å (**Figure 2C**). These hydrodynamic parameters match well those obtained from the crystallographic structure (*R*_g_ 32.5 Å, *D*_max_ 99.6 Å). Indeed, direct comparison of the theoretical scattering calculated with *CRYSOL*, and the experimental scattering confirmed the excellent agreement with a *χ*^2 ^= 1.5 (**Figure 2A**). *Ab initio* shape reconstruction of *Li*GAPDH using dummy-bead models as implemented in *DAMMIF* resulted in a family of volumes with a consistent shape and a calculated resolution for the consensus reconstruction of 28.3 Å (**Supplementary Figure 3**). Attempts to rigid-body fit the crystallographic model of *Li*GAPDH into the *ab-initio* SAXS shape showed a close agreement between the crystal and solution structures (**Figure 2D**). From these observations, we concluded that the overall organization of *Li*GAPDH in solution is preserved in the crystal lattice.

**Table 2:** Small-angle X-ray scattering (SAXS) parameters.

SAXS parameters	
Molecular mass *M* from composition (Da)^1,2^	36 648 (*p.s.*)
Molecular mass *M* for a tetramer (Da)	146 592
Guinier analysis	
*R*_g_ (Å)	35.70 ± 0.09
Quality-of-fit parameter (*r*^2^ fit)	0.84
*P*(*r*) analysis	
*R*_g_ (Å)	34.79 ± 0.03
*D*_max_ (Å)	90.9
*M* (Da) from *I(0*) (ratio to expected value)	143 866 (0.98)
Volume (*V*_P_/*V*_C_)	264 297/744
Structural modeling	
Symmetry/anisotropy assumptions	*P*222/unknown
*χ*^2^ value/range	0.957−0.976
Model resolution (Å)	28.3

^1^ ProtParam, Expasy web server at https://web.expasy.org/cgi-bin/protparam/protparam.

^2^ Theoretical molecular mass calculated from the primary sequence (p.s.).

### 3.3 Active site of *Li*GAPDH

**Figure 3:**
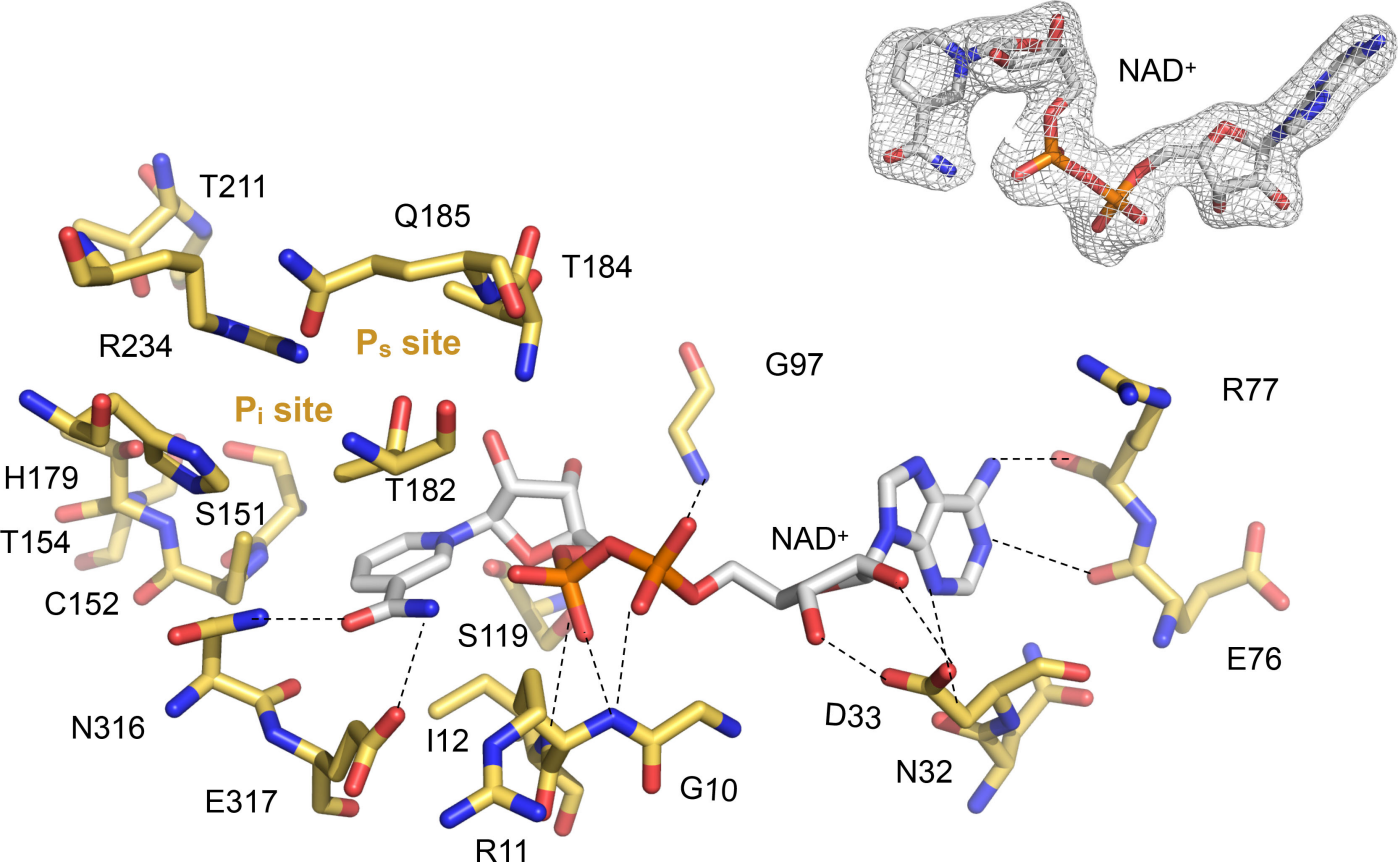
Close-up into the active site of *Li*GAPDH. Catalytic residues are shown in sticks in CPK colors (non-C atoms) or gold (C atoms). The NAD^+^ cofactor is shown in sticks in CPK colors. Dashed lines represent polar interactions (H-bonds). The P_s_ and P_i_ sites are annotated. Inset (top right) shows the quality of the experimental electron density surrounding the cofactor.

The active site of *Li*GAPDH is a large cavity covered by a lid spanning about 50 amino acid residues (residues 114-164). At the bottom of the groove, the NAD^+^ cofactor occupies an elongated binding site between the central β-sheet of the *N*-terminal domain and helices H1-H5, where it makes contacts with the main-chain atoms of Asn32 (from β2), Glu76, and Arg77 (from the β5-3_10_A loop) (**Figure 3**). The catalytic Cys152 residue is located at the intersection between the *N*-terminal and *C*-terminal domains, where it interacts with the side chains of His179 and Arg234, the two other catalytic triad residues, responsible for lowering the p*K*_a_ of the Cys152 thiol nucleophile. Arg234, in turn, interacts with Thr182 and Gln185. *Li*GAPDH lacks an aspartic acid residue between Thr182 and Gln185, unlike other GAPDH sequences like those of *C. perfringens* and *S. pyogenes* (19).

In contrast to other GAPDH structures, in *Li*GAPDH, well-ordered electron density was found for the residues responsible for binding the inorganic phosphate moieties of substrates and products, the so-called P_s_ and P_i_ binding sites (**Figure 3**). Inspection of the eight active sites in the crystal structure revealed that some of them had the P_s_ and P_i_ sites occupied by phosphate anions from the purification buffers or glycerol molecules from the cryoprotectant solution, which acted as substrates or substrate analogs. These ligands corroborated the relevance of the observed active-site configuration, which remained unchanged with or without substrate analogs across all subunits. The P_s_ site is formed by residues Thr182, Thr184, and Gln185, and the P_i_ site by residues Ser151, Thr153, His179, Thr211, and Gly212. The catalytic triad residue Arg234 interacts with both phosphate sites, thereby it belongs to the P_s_ and P_i_ sites.

### 3.4 *Li*GAPDH kinetic parameters

Besides carrying out moonlighting functions in the extracellular space, *Li*GAPDH is a glycolytic enzyme located in the cytoplasm. We have measured its enzymatic activity using a well-established assay that reports on the reduction of the NAD^+^ cofactor to NADH at 25 °C and pH 8.5 (18, 19). In these assays, the kinetic parameters for the direct reaction catalyzed by *Li*GAPDH were determined by systematically varying NAD^+^, G3P, and P_i_ concentrations. While we observed a classic hyperbolic dependence of the catalytic activity for G3P/P_i_ at low substrate concentration (Michaelis-Menten model), with excess of either G3P or P_i_ the enzyme exhibited significant substrate inhibition with inhibitory constants *K*_SS_ larger though within one order of magnitude of the corresponding *K*_m_ values (**Supplementary Figure 4**). Kinetic parameters are reported in **Table 3**.

**Table 3:** *Li*GAPDH kinetic parameters.

	*V*_max_ (m*M* min^–1^)	*K*_m_ (m*M*)	*K*_SS_ (m*M*)	*k*_cat_ (s^–1^)	*k*_cat_/*K*_m_ (m*M*^–1^ s^–1^)
G3P	(4.2 ± 0.4) × 10^–2^	0.6 ± 0.1	2.0 ± 0.3	70 ± 7	121 ± 24
NAD^+^	(2.65 ± 0.05) × 10^–2^	0.081 ± 0.006	–	44.2 ± 0.9	543 ± 44
P_i_	(6.6 ± 0.4) × 10^–2^	1.2 ± 0.1	10 ± 1	110 ± 7	91 ± 13

### 3.5 Inhibition of *Li*GAPDH by two natural compounds

Next, we examined the inhibitory effect of two natural compounds, anacardic acid and curcumin, which have previously shown efficacy against GAPDH from the Gram-positive bacterial pathogens *A. vaginae* (18) and *S. pyogenes* (19), and, in the case of anacardic acid, *Trypanosoma cruzi* (52). The safety of these compounds for use in humans makes them attractive lead compounds in repositioning campaigns for leptospirosis.

**Figure 4:**
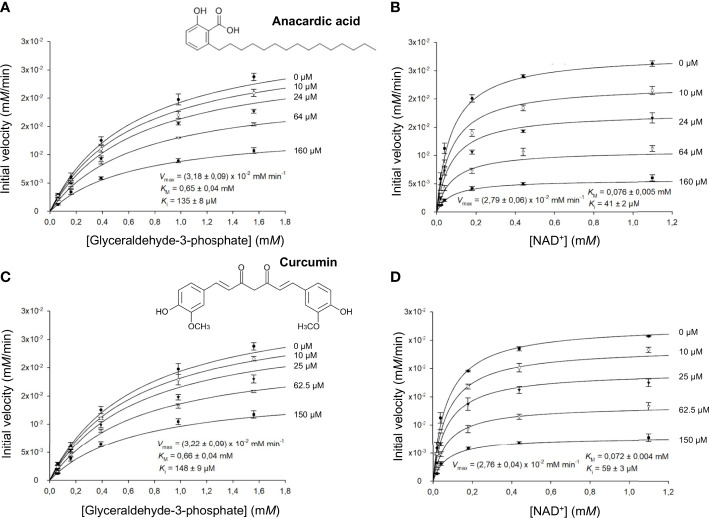
*LiGAPDH* can be inhibited by natural products. Initial rate of reaction plotted in the presence of various concentrations of anacardic acid (10-160 µ*M*) against the concentration of **(A)** glyceraldehyde-3-phosphate (G3P) or **(B)** NAD^+^, and in the presence of curcumin (10-150 µ*M*) against the concentration of **(C)** glyceraldehyde-3-phosphate (G3P) or **(D)** NAD^+^. Each experimental data point represents the mean and the errors are standard deviations of the mean (SEM) from three independent experiments. Nonlinear regression to a Michaelis-Menten hyperbolic model with noncompetitive inhibition was carried out with SigmaPlot 14.5 (*R*^2^ = 0.98).

We obtained comparable results after testing the two natural compounds in the range 10-160 μ*M* (anacardic acid) and 10-150 μ*M* (curcumin) while varying the concentrations of either the G3P substrate or the NAD^+^ cofactor (**Table 4**; **Figure 4**; **Supplementary Figures 5**, **6**). Both anacardic acid and curcumin behaved as micromolar non-competitive inhibitors of *Li*GAPDH with respect to G3P and NAD^+^. Not only was the inhibitory modality the same for both natural products, but also the magnitude of the inhibition constants was comparable: *K*_i_^ana/G3P^ = 135 μ*M vs. K*_i_^cur/G3P^ = 148 μ*M* and *K*_i_^ana/NAD^ = 41 μ*M vs. K*_i_^cur/NAD^ = 59 μ*M*.

**Table 4:** Inhibition of *Li*GAPDH by anacardic acid and curcumin.

Inhibitor	Mode	Substrate	*V*_max_ (m*M* min^–1^)	*K*_m_ (m*M*)	*K*_i_ (µ*M*)	*k*_cat_ (s^–1^)	*k*_cat_/*K*_m_ (m*M*^–1^ s^–1^)
Anacardic acid	NC^1^	G3P	(3.18 ± 0.09) × 10^–2^	0.65 ± 0.04	135 ± 8	53 ± 1	82 ± 6
		NAD^+^	(2.79 ± 0.06) × 10^–2^	0.076 ± 0.005	41 ± 2	46.5 ± 0.9	612 ± 43
Curcumin	NC	G3P	(3.22 ± 0.09) × 10^–2^	0.66 ± 0.04	148 ± 9	54 ± 2	81 ± 6
		NAD^+^	(2.76 ± 0.04) × 10^–2^	0.072 ± 0.004	59 ± 3	46 ± 1	637 ± 33

^1^ NC, noncompetitive inhibition.

### 3.6 Interaction between C5a and *Li*GAPDH

**Figure 5:**
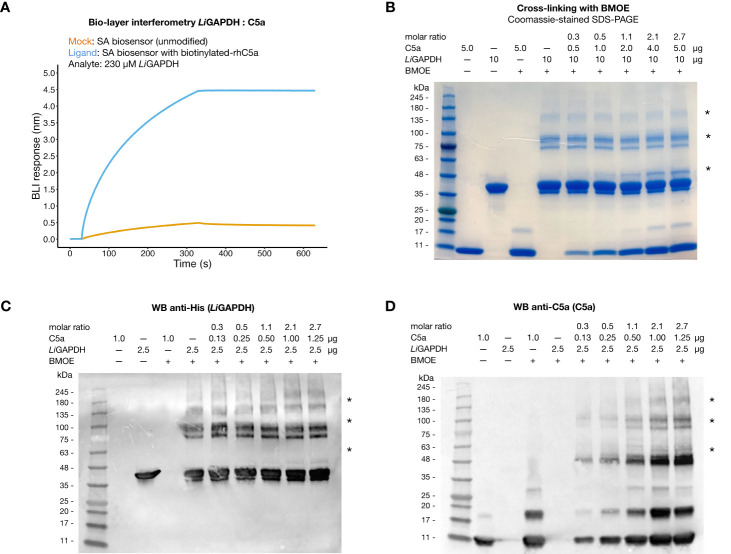
Interaction of *Li*GAPDH-C5a. **(A)** Bio-layer interferometry sensorgrams showing the wavelength shift length or BLI response (nm) obtained by incubating streptavidin (SA) biosensors previously loaded with biotinylated-C5a (blue line) or unmodified (orange line) with 230 µ*M Li*GAPDH. **(B)** SDS-PAGE electrophoretic separation of cross-linking reactions of *Li*GAPDH and C5a with BMOE. Gel loaded with mock-treated control samples and BMOE-treated samples (increasing concentrations of C5a for a fixed concentration of *Li*GAPDH). The first two lanes with added BMOE represent internal controls for C5a (maximum load) and *Li*GAPDH without added C5a. **(C)** Western blotting of the same samples in **(A)** revealed with an anti-His HRP antibody. **(D)** Like **(C)** with an anti-C5a primary antibody **(D)**. Asterisks indicate protein bands containing cross-linked *Li*GAPDH:C5a complexes.

It has been reported that C5a can interact with immune evasive factors like GAPDH on the pathogen’s surface through weak, transient interactions, which might be enhanced by the high local concentration of surface associated GAPDH (16, 18, 19). To reveal a potential interaction between natively folded *Li*GAPDH and C5a *in vitro*, we performed bio-layer interferometry experiments with streptavidin (SA) biosensors loaded with biotinylated-C5a. At a high *Li*GAPDH concentration (230 µ*M*, ~9 mg/mL), we observed a clear binding event with immobilized C5a characterized by a low kinetic association constant (*k*_on_ estimated at 37.3 ± 0.2 *M*^–1^ s^–1^) and a slow dissociation constant, which could not be accurately determined (**Figure 5A**). Binding to the unmodified SA biosensors was far lower at the same concentration of *Li*GAPDH, ruling out strong nonspecific interactions between the biosensor and the analyte.

To provide independent evidence for the interaction, we resorted to the highly specific cross-linker reagent BMOE. BMOE covalently tethers the free sulfhydryl groups of Cys residues that lie in close proximity, typically at a distance of 8 Å apart. This stringent condition discriminates nonspecific interactions from meaningful though weak interactions, at the risk of missing some authentic but little populated complexes or those that would have required a longer tether. As shown in **Figure 5B**, SDS-PAGE electrophoresis of BMOE-treated mixtures of *Li*GAPDH and C5a revealed the appearance of a band corresponding to cross-linked *Li*GAPDH-C5a complexes, clearly discernable already at substoichiometric C5a:*Li*GAPDH molar ratios (between 0.53-1.07). The molecular mass of this cross-linked band (~10 kDa greater than monomeric *Li*GAPDH) coincided with the expected mass increment due to C5a (molecular mass ~9 kDa) (**Figure 5B**, indicated by an asterisk). The intensity of this band depended on the amount of C5a in the cross-linking reaction, thus proving that a native, though weak interaction is likely to exist between *Li*GAPDH and C5a. We could identify proteins bands simultaneously containing C5a and hexahistidine-tagged *Li*GAPDH by Western blotting with anti-C5a and anti-His antibodies (**Figures 5C, D**, indicated by asterisks), demonstrating the formation of *Li*GAPDH:C5a cross-linked complexes. This interaction necessarily involves protein surfaces containing a free Cys residue. The only free Cys in C5a (Cys704 in C5 numbering, Cys27 in C5a numbering) must therefore mediate this interaction. Based on previous evidence (16) we hypothesize that the catalytic Cys residue (Cys152 in *Li*GAPDH) participates in this interaction. Cys152 is the most reactive Cys residue in *Li*GAPDH since the nucleophilicity of its thiol moiety is enhanced by the catalytic environment. The specificity of the reaction was corroborated by the observation that no cross-linked bands developed in samples containing only C5a until later than the *Li*GAPDH-C5a cross-linked complexes, nor did they spontaneously appear on samples not treated with BMOE (**Figure 5B**).

**Figure 6:**
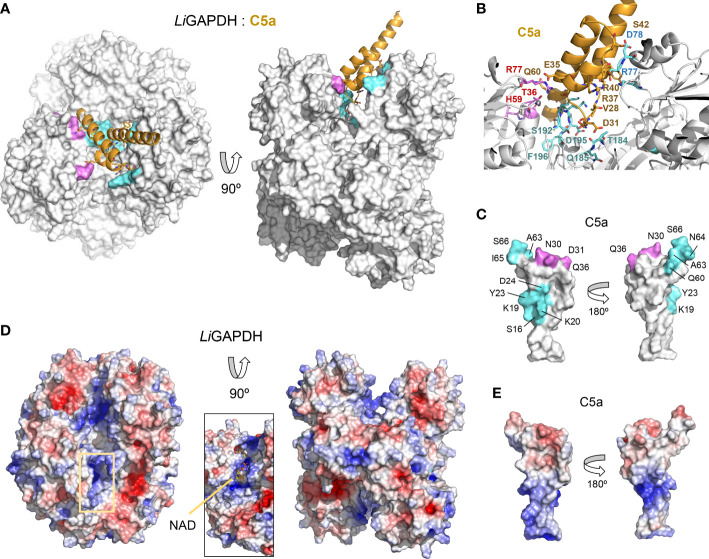
*Li*GAPDH-C5a complex by guided docking. **(A)** Two views 90° apart of the top-ranking *Li*GAPDH-C5a docking pose, shown in molecular surface representation (*Li*GAPDH, in white) and in cartoon (C5a, in orange). *Li*GAPDH residues engaged in polar interactions with C5a are shown in aquamarine (Q subunit) or violet (P subunit). **(B)** Zoom into the binding interface with interacting residues shown in sticks and annotated, color coded as in **(A)**. **(C)** Two orientations 180° apart of C5a shown in molecular surface representation, with residues engaged in interactions with the Q and P subunits of *Li*GAPDH colored in aquamarine and violet, respectively. **(D)** Electrostatic potential surface of *Li*GAPDH mapped onto the same two orientations shown in **(A)**. The inset zooms into the NAD^+^ binding site, highlighting the positively charged residues in the neighboring area. **(E)** Electrostatic potential surface of C5a mapped onto the same two orientations shown in **(C)**. It can be noted that the region around Cys27 is slightly negatively charged.

To shed light on the structure of the *Li*GAPDH-C5a binary complexes, we carried out cross-link guided protein-protein docking with *ROSETTA*, as distance restraints from chemical cross-linking experiments can guide protein-protein docking calculations and significantly improve the accuracy of the simulations (45). All the calculated docking poses were clustered and filtered according to binding energy, compliance with distance restraints derived from the cross-linker, and the buried surface area. After analyzing the 10 most populated clusters of binary complexes (**Supplementary Figure 7**), the most likely docking pose was identified (**Figure 6A**). In this pose, C5a approximates the *Li*GAPDH deep groove at the interface between chains Q and P, making polar interactions with residues from both chains (shown in aquamarine and violet, respectively, in **Figures 6A, B**). Residues from C5a implicated in binding include residues near Cys27 (residues 23-36) and also from the *N*- and the *C*-terminal helices (**Figure 6C**, color coded as in **Figures 6A, B**). As far as the electrostatic potential is concerned, the face of C5a that comes closest to *Li*GAPDH bears a slightly negative charge, which is complementary to the long and positively charged cavity of the active site and neighboring residues (**Figures 6D, E**). The *C*-terminal helix of C5a tilts at an angle that allows it to slide out of the binding site. This orientation is consistent with known facts about the interaction of GAPDH from various organisms with mostly α-helical proteins of small size (53–55).

**Figure 7:**
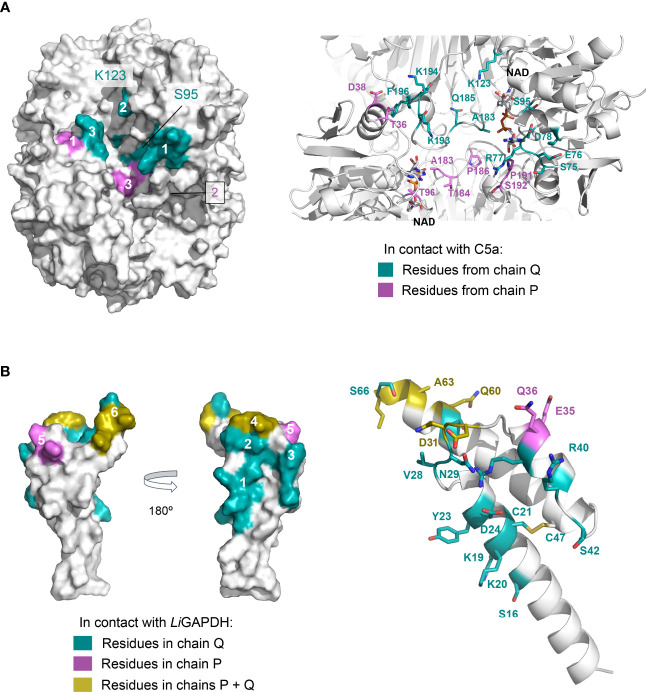
Consensus docking surfaces for *Li*GAPDH-C5a. **(A)** Molecular surface representation of *Li*GAPDH (in white). Surface areas that interact with C5a in most of the top-ranking docking poses (consensus docking surfaces) are highlighted in aquamarine if the residues come from the Q subunit, and violet if they come from the P subunit. Spatially close residues define up to three interaction patches on chains Q and P. The inset shows a zoom into the area containing the consensus docking residues (shown in sticks with the same color coding); *Li*GAPDH is shown in cartoon (in white). **(B)** Likewise, for C5a. Two orientations 180° apart are shown for the molecular surface representation. Residues in aquamarine interact with the Q subunit and in violet with the P subunit of *Li*GAPDH; the few residues that interact with both the Q and P subunits are colored yellow. Patches are numbered 1 through 6. Patches 4 and 6 (in yellow) pivot between binding the Q or the P subunits depending on the specific pose. In addition, C5a is shown in cartoons with the residues predicted to interact with *Li*GAPDH shown in sticks and with the same color coding.

Other high-ranking docking poses exploited the same or very similar docking surfaces as the top-ranking pose, differing mainly in the angle with which C5a approximated *Li*GAPDH (**Supplementary Figure 7**). Using the most representative poses, which differ minimally in the binding site, we can describe a consensus binding interface (**Figure 7**). Concerning *Li*GAPDH, the surface residues that mediate most contacts with C5a belong to the *N*- and *C*-terminal domains and to two chains, Q and P. On subunit Q, the three identified patches comprise residues 75-81 (patch 1Q), 183-185 (patch 2Q), and 192-196 (patch 3Q); and, on subunit P, residues 36-38 (patch 1P), 7 and 96-97 (patch 2P), and 182-186 and 191-196 (patch 3P) (**Figure 7A**). Reciprocally, the C5a residues that consistently contributed to the docking interface in the top-ranking docking solutions comprised residues surrounding Cys27 and from the two long helices (**Figure 7B**); the end of the *C*-terminal helix was pointing out of the docking site in most poses. The generally polar nature of the interface of most docking poses and the relatively wide range of compatible tilts for C5a (within the same small docking area) are compatible with a weak interaction between C5a and *Li*GAPDH, which could be enhanced in the biological context through electrostatic and avidity effects as previously described for GAPDH from Gram-positive pathogens (18, 19).

## 4 Discussion

Vertebrate innate immunity has evolved to prevent and fight infections by recognizing features of pathogens that are broadly shared such as plasma membrane lipids and cell-wall composition, called pathogen-associated molecular patterns (PAMPs). The complement system is one of the oldest and most efficient branches of our innate immunity. It therefore makes evolutionary sense that many pathogens have evolved sophisticated molecular weaponry to circumvent, inactivate, or mimic components of the complement system. Bacteria (and many eukaryotic parasites) rely on multiple complement-evasive strategies, often deployed simultaneously. One of these complement-targeting strategies consists in the neutralization of the C5a anaphylatoxin, which bacteria have learned to do in two separate but complementary ways: by proteolytic inactivation and by direct binding (sequestration). Proteases that can cleave C5a are deployed by pathogenic bacteria (56) as diverse as *Pseudomonas aeruginosa* (the alkaline protease ArpA and elastase B LasB) (57) and all sequenced serotypes of Group B streptococci (streptococcal cell-wall C5a peptidase) (58, 59).

Besides the direct proteolytic cleavage of C5a, some pathogens have also evolved the capacity to bind and retain C5a close to the site of infection, thereby precluding the anaphylatoxin (or, at least, slowing it down) from recruiting neighboring phagocytes. One of the virulence factors is the moonlighting protein GAPDH. Intracellularly, GAPDH is the well-known glycolytic enzyme; however, outside the cell, GAPDH can bind C5a (and, in some microorganisms, C3). In *S. pneumoniae*, GAPDH remains associated with the cell wall, indicating that bound C5a remains attached to the pathogen cells (16). Reciprocally, in clinical isolates of *S. pyogenes*, added C5a binds the cells in a dose-dependent fashion (19). These two complementary views show that streptococcal cells have the ability to “soak in” C5a, effectively shielding it from macrophages. Other cell-wall components might help to retain C5a besides GAPDH, further enhancing the immune evasive effect.

*L. interrogans* is a Gram-negative pathogen with an impressive array of immune evasive mechanisms, including many targeted at complement factors (13, 60). Several of the best characterized immune evasion mechanisms of *L. interrogans* include recruiting endogenous complement regulators such as FH and C4BP (13, 61, 62). However, complement-targeting immune evasion mechanisms directly interfering with C5a had not been demonstrated. In this work, we have shown that C5a binding and sequestration through *Li*GAPDH can provide an additional immune evasive mechanism to an already impressive weaponry. In support of this view, other metabolic and glycolytic enzymes have been shown to perform similar functions, such as enolase (22), EF-Tu (63), and the chaperonin GroEL (64). The *in vivo* relevance of these interactions for the pathogen’s survival in the host is still a matter of debate, further complicated by the essential nature of the bacterial genes encoding most moonlighting proteins, which precludes the analysis of gene deletion phenotypes, and the high concentrations found for these proteins in both the bacterial cytosol and exoproteome. Another difficulty for dissecting the relevance of specific moonlighting/virulence factors arises from the multiplicity of redundant and nonredundant immune evasive strategies that appear to contribute to the adaptation of *L. interrogans* and other bacterial pathogens to their hosts.

To better understand the unconventional roles of *Li*GAPDH, we have solved the crystal structure of the holoenzyme to 2.37-Å resolution. The crystallographic structure agrees with the solution SAXS data, suggesting that the crystal lattice has trapped the native conformation. The structural information it provides contributes to a significant pool of GAPDH structures available at the PDB. This information can be used for purposes such as drug discovery and repurposing campaigns in cases where GAPDH has a role in completely expressing pathogen’s virulence. This unconventional role appears to be rather prevalent as phylogenetically diverse Gram-negative and Gram-positive bacteria (and at least one eukaryotic parasite too) exhibit it (16, 18–21, 56). In this light, the kinetic characterization that we have performed on *Li*GAPDH shows that the enzyme is susceptible to inhibition by natural products such as anacardic acid and curcumin. Given the favorable safety profile of these natural products, they represent promising starting points for further drug development.

In the context of leptospirosis, extracellular *Li*GAPDH may play a virulence role by binding to C5a generated by the activation of complement’s terminal pathway. Indeed, *Li*GAPDH has been shown to be one of the twenty most abundant proteins in the extracellular proteome of pathogenic *L. interrogans* strains (11). Although not yet known, *Li*GAPDH could be exported to the cellular exterior by type I or II secretion systems or *via* extracellular vesicles, two of the most common secretion mechanisms characterized in *L. interrogans*.

In this work, we have shown by bio-layer interferometry and cross-linking experiments that *Li*GAPDH and C5a can form a specific complex at a sufficiently high concentration to overcome an intrinsically slow kinetic association constant. Initially, a weak interaction between nascent C5a (generated *in situ* on the surface of opsonized bacteria) and *Li*GAPDH could delay C5a diffusion long enough to be proteolytically degraded by nonspecific proteases from the pathogen or dearginated by serum carboxypeptidases; in fact, deargination of anaphylatoxins C5a and C3a *in vivo* is a fast and irreversible process that dampens the chemotactic response. In either scenario, neutrophil recruitment to the site of infection would be much reduced. The cross-link guided docking protocol that we have explored in this work produces *Li*GAPDH-C5a complexes that are compatible with the known facts about the interaction: proximity between C5a Cys27, the only free Cys residue in C5a, and the highly reactive catalytic Cys152 residue; structural and electrostatic complementarity at the docking site; a considerable buried interface (>2000 Å^2^); a predominantly electrostatic nature; and a variety of compatible poses differing in the overall tilt of C5a inside the interfacial groove between *Li*GAPDH Q-P subunits.

As our structural knowledge of the molecular machinery of the host’s innate immunity and the pathogens’ immune evasion factors expands and refines, our tools to fight recalcitrant infections will likely become more efficient and sophisticated. In the face of the dwindling efficacy of antibiotics and the looming medical and humanitarian crisis unleashed by global warming, further research is sorely needed to generate new approaches to curb infectious diseases through the combination of structural information from the host’s innate immune system and the pathogens’ virulence factors.

## Data availability statement

The datasets presented in this study can be found in online repositories. The names of the repository/repositories and accession number(s) can be found in the article/**Supplementary Material**.

## Author contributions

FJF and MCV contributed to the conception of the study and the design of all the experiments. SNY and KdlP expressed and purified the protein samples for biochemical and structural analysis. SNY, KdlP, and SGQ performed kinetics experiments. SNY performed the BLI experiments. JQG carried out the cross-linking experiments, FJF and MCV ran the cross-link guide docking calculations and analyses. FJF and MCV collected the diffraction data. SNY, FJF and MCV solved and refined the crystal structure and the SAXS shape. FJF and MCV wrote the manuscript. SRdC read the manuscript critically. All authors contributed to the article and approved the submitted version.
