# Supplementary material for: The structure of Leptospira interrogans GAPDH sheds light into an immunoevasion factor that can target the anaphylatoxin C5a of innate immunity
Source: Front Immunol. 2023 Jun 20;14:1190943. doi: 10.3389/fimmu.2023.1190943 (PMC10318897; doi:10.3389/fimmu.2023.1190943)
Supplement: Supplementary file 1 [file DataSheet_1.pdf]

Supplementary Material

The structure of *Leptospira interrogans* GAPDH sheds light into an immunoevasion factor that can target the innate immunity anaphylatoxin C5a

Sergio Navas-Yuste[1], Karla de la Paz[1,2], Javier Querol-García[1,2], Sara Gómez-Quevedo[1,3], Santiago Rodríguez de Córdoba[1,4], Francisco J. Fernández[1,2*] and M. Cristina Vega[1*]

*** Correspondence:** M. Cristina Vega: cvega@cib.csic.es. Francisco J. Fernández: fjfernandez@abvance.com.

1 Supplementary Figures and Tables

This Supplementary Material file contains the following tables and figures:

Supplementary Figure 1. Interface between the O and P chains.

Supplementary Figure 2. Interface between the O and R chains and between the three chains O, P, and R.

Supplementary Figure 3. Representative shapes of *Li*GAPDH restored from SAXS by *ab initio* bead modeling.

Supplementary Figure 4. Kinetic parameters of *Li*GAPDH.

Supplementary Figure 5. *Li*GAPDH inhibition by anacardic acid.

Supplementary Figure 6. *Li*GAPDH inhibition by curcumin.

Supplementary Figure 7. Top-ranking docking sites for C5a on *Li*GAPDH.

Supplementary Table 1. Small-angle X-ray scattering (SAXS) parameters.

1.1 Supplementary Figures

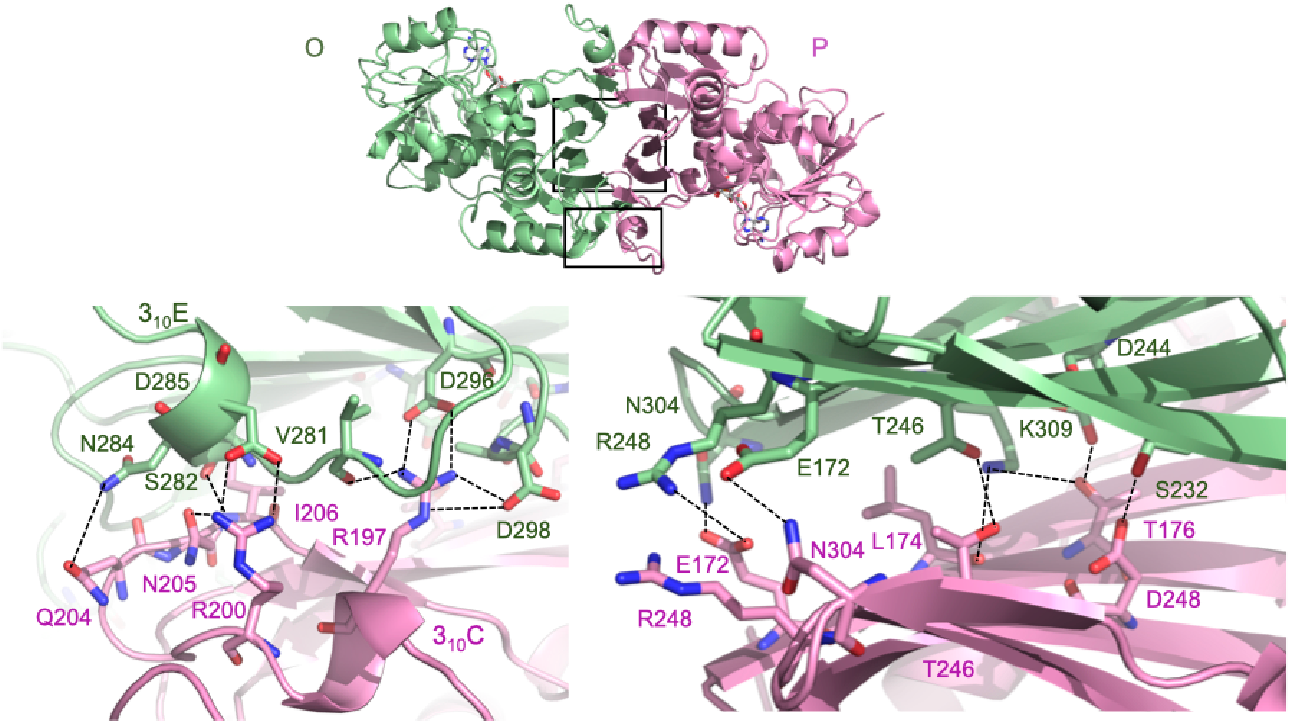

Supplementary Figure 1. Interface between the O and P chains. Cartoon representation of the O and P chains of *Li*GAPDH, color coded as in Figure 1 of the main text. The largest interaction surface between neighboring subunits in *Li*GAPDH involve the O and P chains. The residues implicated in this interface come from the C-terminal catalytic domain: β-strands β8 and β16, helices H6-H8 and 3_{10}C-3_{10}E. Most interactions at this interface have a polar/charged character. The buried surface area ranges from 1886 to 1907 Å^2 with an average of 1901 Å^2. Up to 19 salt bridges and 9 H-bonds stabilize the interaction. For example, Arg197 from helix 3_{10}C forms a salt bridge with Asp296; Arg200 with two aspartic acid side chains: Asp285 (3_{10}E) and Asp298 (β10). The two-fold symmetry between the O and P chains duplicates the interactions. Some of the H-bonds at this interface are: Arg197 with Val281; Asp244 with Thr176; Ser283 with Ile206; and Asn284 and Asn204 with Asn205.

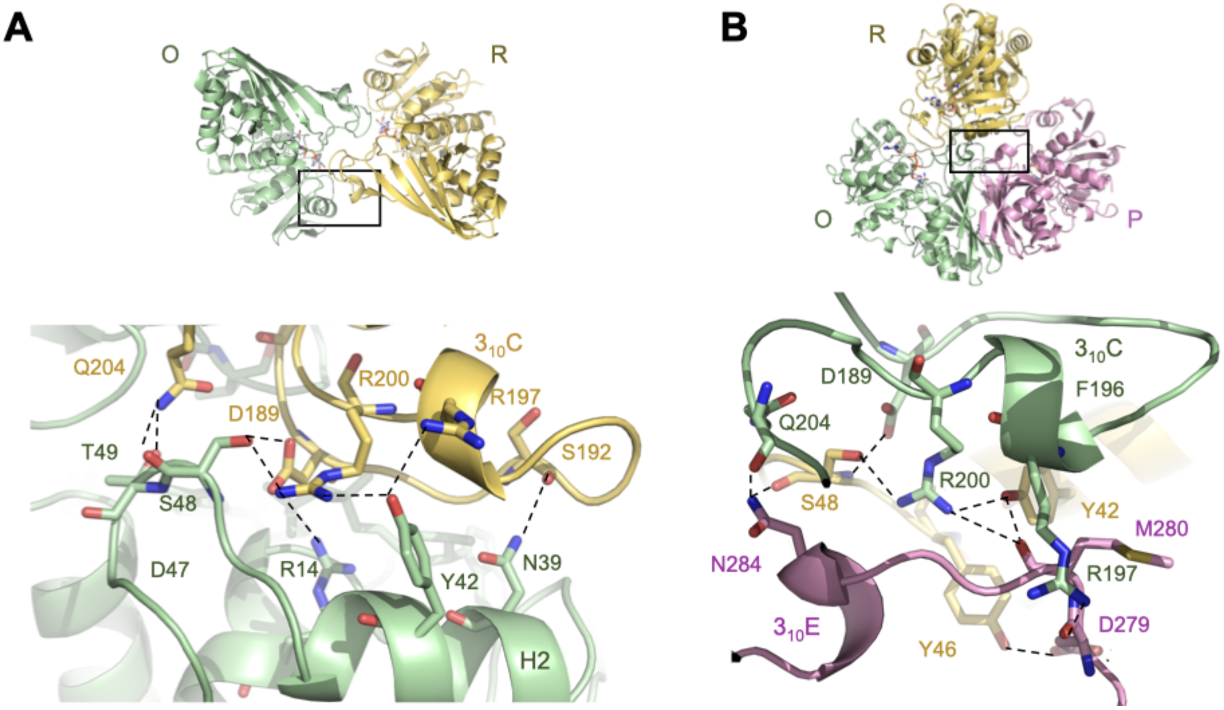

Supplementary Figure 2. Interface between the O and R chains and between the three chains O, P, and R. (A) Cartoon representation of the O and R chains of *Li*GAPDH, color coded as in Figure 1 of the main text. The buried surface area at the O-R interface ranges between 1400 and 1421 Å^2 with an average of 1411 Å^2. A total of 12 H-bonds stabilizes this interface. The H-bonds involve the following residue pairs: Arg14 with Asp189; Ser48 with Asp189; Thr49 with Gln204; Thr187 with Thr182; Ser192 with Asn39; Arg2022 with Ser48; Asp189 with Ser48; and, due to the two-fold symmetric interactions, the same interactions occur between the symmetric residues. **(B)** Cartoon representation of the O, P, and R subunits of *Li*GAPDH, color coded as in Figure 1 of the main text. The buried area at this interface ranges from 484 to 505 Å^2 with an average of 492 Å^2. Ten H-bonds and 2 salt bridges contribute to stabilizing this interface: Tyr42 with Asp279 and Met280; Arg52 with Asp285; and Asn284 with Ser48; due to the two-fold symmetric interactions, the same set of interactions occur between the symmetric residues.

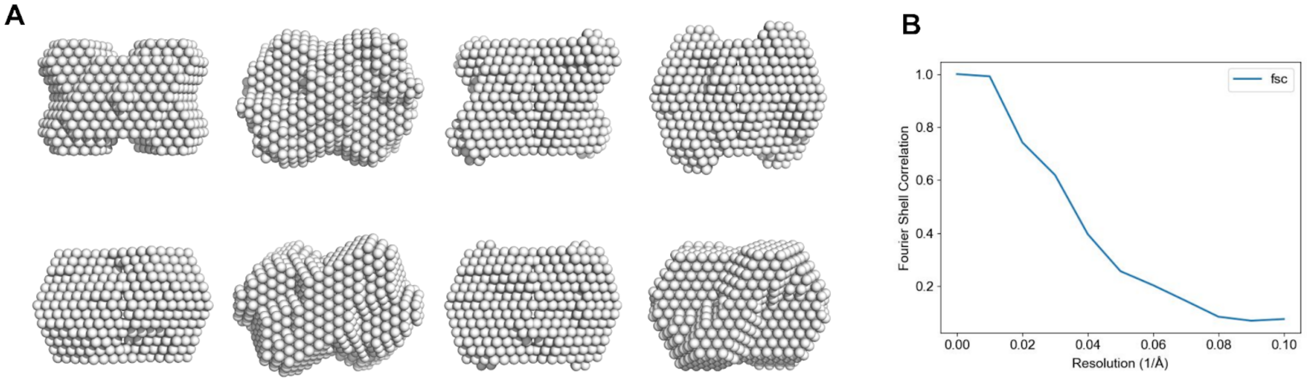

Supplementary Figure 3. Representative shapes of *Li*GAPDH restored from SAXS by *ab initio* bead modeling and resolution. (A) Most representative (non-similarity distance, NSD = 0.55) bead models obtained from SAXS *ab initio* calculations with *DAMMIF* (1). **(B)** Resolution (28 Å) of the SAXS reconstructions estimated from the Fourier shell correlation (FSC) curve at FSC = 0.5. This estimate includes all the bead models obtained, not only the most representative models; when only the most representative bead models are analyzed, the resolution of the reconstructed models is 27 Å.

1. Franke D, Svergun DI. DAMMIF, a program for rapid ab-initio shape determination in small-angle scattering. *J Appl Crystallogr* 2009 Apr 1;42(2):342–6. doi:10.1107/S0021889809000338

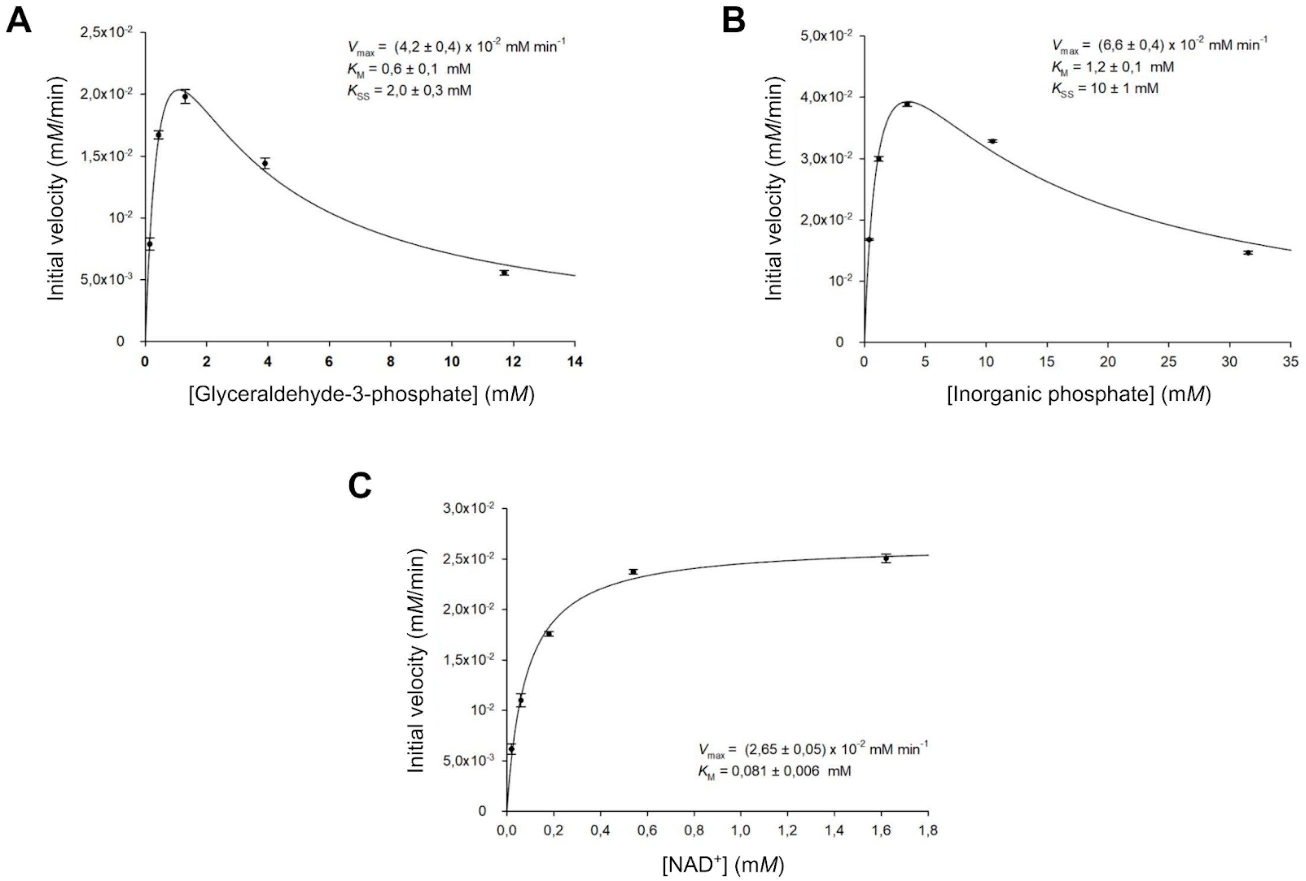

Supplementary Figure 4. Kinetic parameters of *Li*GAPDH. (A) Initial rate of reaction *versus* the substrate glyceraldehyde-3-phosphate (G3P). **(B)** Initial rate of reaction *versus* the substrate inorganic phosphate (P_i). **(C)** Initial rate of reaction *versus* the NAD^+ cofactor (NAD^+). Methods are described in the main text. Kinetic parameters V_{max}, K_m, and, if applicable, K_{SS} (the K_i for substrate inhibition) are annotated on each plot. Substrate inhibition is strong for G3P and P_i, but it is absent for NAD^+ at the tested concentration range.

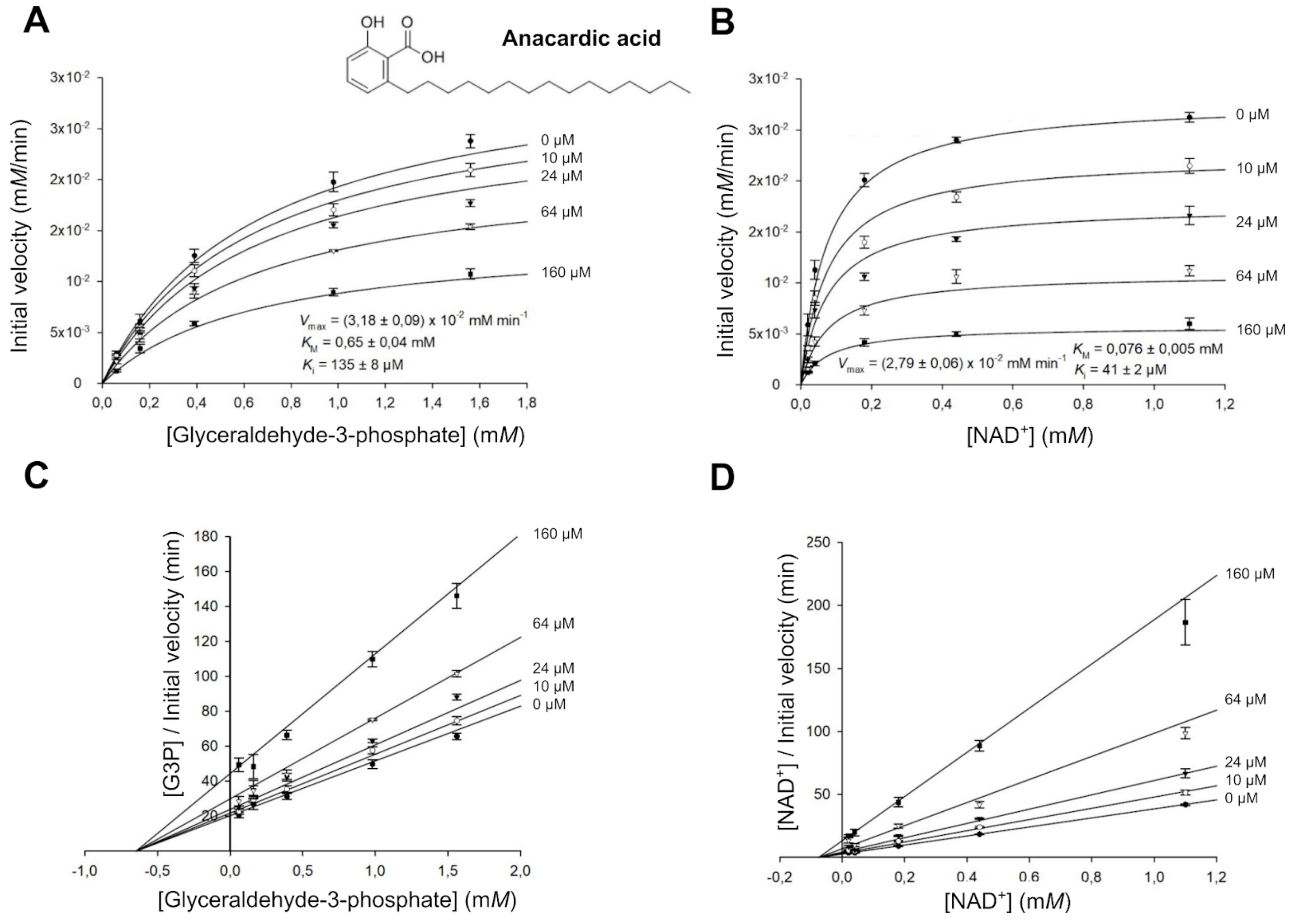

Supplementary Figure 5. *Li*GAPDH inhibition by anacardic acid. (A) Initial rate of reaction *versus* the substrate glyceraldehyde-3-phosphate (G3P) at various concentrations of anacardic acid (10-160 μ*M*). **(B)** Initial rate of reaction *versus* the NAD^+ cofactor (NAD^+) at various concentrations of anacardic acid (10-160 μ*M*). Panels (A) and (B) together are reproduced in the main text as Figure 4A. **(C)** Same data in (A) replotted as a Hanes-Woolf plot to determine the inhibition modality of anacardic acid with respect to G3P (noncompetitive inhibition in this case). **(D)** Same data in (B) replotted as a Hanes-Woolf plot to determine the inhibition modality of anacardic acid with respect to NAD^+ (noncompetitive inhibition in this case). Each experimental data point represents the mean and the errors are standard deviations of the mean (SEM) from three independent experiments. Nonlinear regression to a Michaelis-Menten hyperbolic model with noncompetitive inhibition was carried out with SigmaPlot 14.5 (R^2 = 0.98).

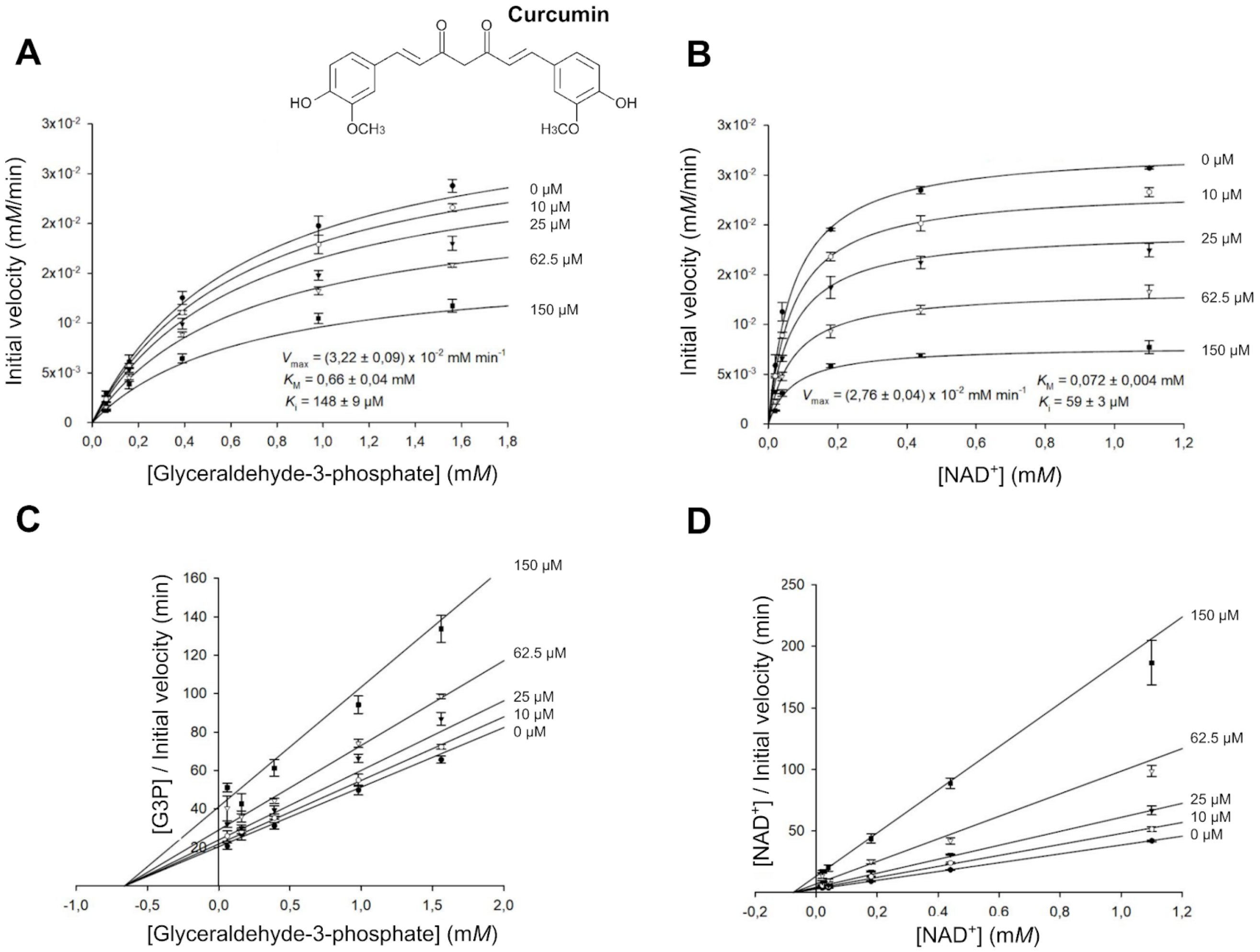

Supplementary Figure 6. *Li*GAPDH inhibition by curcumin. (A) Initial rate of reaction *versus* the substrate glyceraldehyde-3-phosphate (G3P) at various concentrations of curcumin (10-150 µ*M*). **(B)** Initial rate of reaction *versus* the NAD^+ cofactor (NAD^+) at various concentrations of curcumin (10-150 µ*M*). Panels (A) and (B) together are reproduced in the main text as Figure 4B. **(C)** Same data in (A) replotted as a Hanes-Woolf plot to determine the inhibition modality of curcumin with respect to G3P (noncompetitive inhibition in this case). **(D)** Same data in (B) replotted as a Hanes-Woolf plot to determine the inhibition modality of curcumin with respect to NAD^+ (noncompetitive inhibition in this case). Each experimental data point represents the mean and the errors are standard deviations of the mean (SEM) from three independent experiments. Nonlinear regression to a Michaelis-Menten hyperbolic model with noncompetitive inhibition was carried out with SigmaPlot 14.5 ($R^2 = 0.98$).

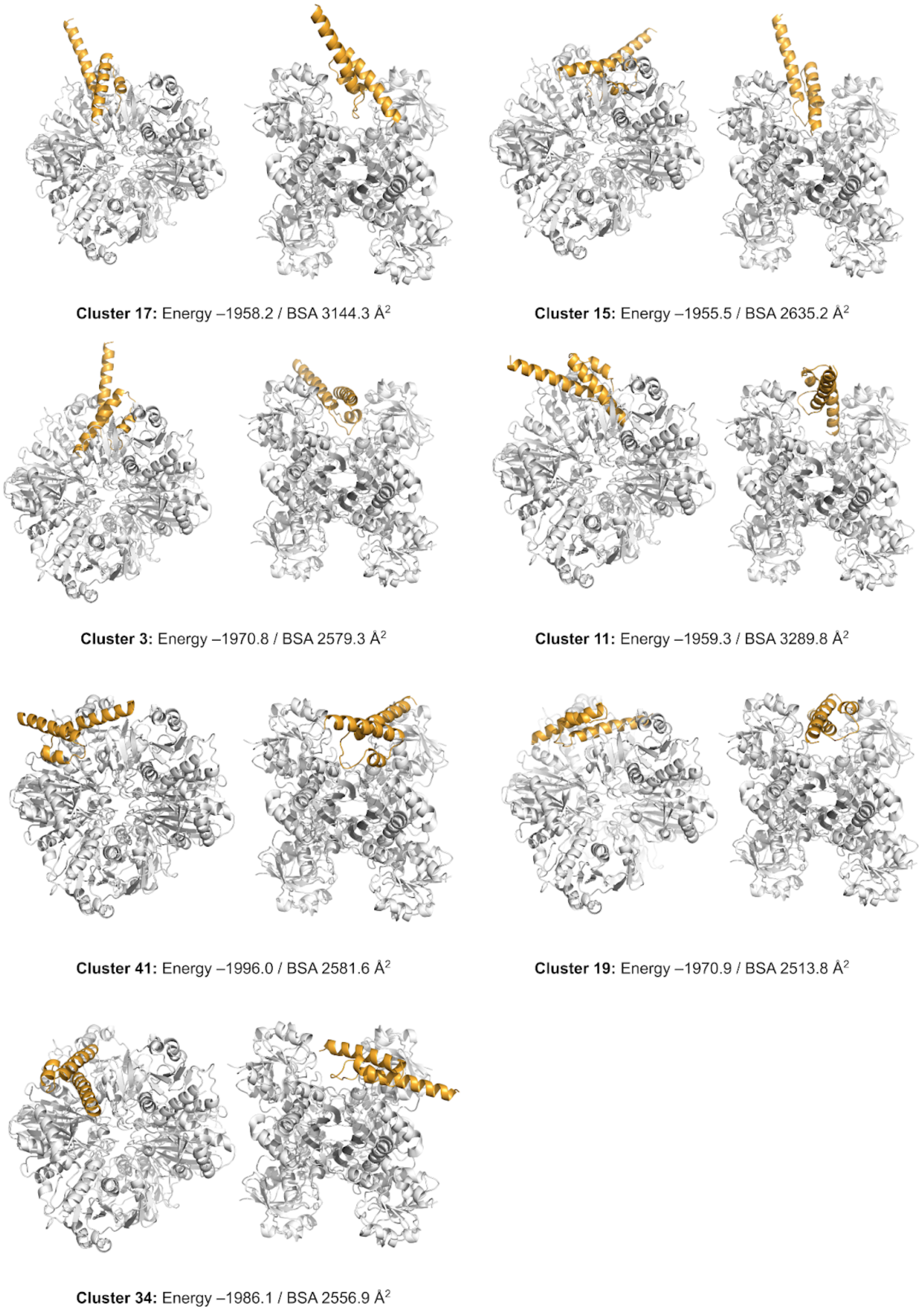

Supplementary Figure 7. Top-ranking docking sites for C5a on *Li*GAPDH. Gallery of the docking poses selected for further analysis based on distance between $Cys152^{GAP}$ and $Cys27^{C5a}$, the *ROSETTA* energy score (“energy”) of the docking calculations, and the buried surface area (BSA) at the interface between C5a and *Li*GAPDH. Proteins are shown in cartoon representation, *Li*GAPDH in white and C5a in orange. While the main docking site is always the same, the tilt of C5a with respect to *Li*GAPDH is variable.

1.2 Supplementary Tables

Supplementary Table 1. Small-angle X-ray scattering (SAXS) parameters.

(*a*) Sample details	
	*Li*GAPDH
Source	Recombinant protein expressed in *E. coli* BL21(DE3)
Description	Uniprot ID Q72QM3 (Q72QM3_LEPIC)
Theoretical extinction coefficient at 280 nm in water ε^{280} (M^{-1} cm^{-1})[1]	28 679 (*ox*)<br>28 420 (*red*)
Molecular mass *M* from composition (Da)[1,2]	36 648 (*p.s.*)
Molecular mass *M* for a tetramer (Da)	146 592
Concentration (range/values) measured	1.25–10 mg/ml
Solvent composition	10 m*M* HEPES-NaOH (pH 7.4), 150 m*M* NaCl, 3.4 m*M* EDTA, 2 m*M* TCEP, 3% (v/v) glycerol
(*b*) SAS data collection parameters	
Source, instrument and description or reference	B21 BioSAXS beamline at Diamond
Wavelength (Å) / energy (keV)	0.94 / 13.1
Sample-to-detector distance (m)	3.7
q-measurement range (Å^{-1})	0.0026–0.34
Method for monitoring radiation damage	Visual inspection of data frames
Exposure time, number of exposures	3 s/frame (620 frames in HPLC mode)
Sample configuration (temperature in ºC)	50 µl (9.0)
(*c*) Software employed for SAS data reduction, analysis and interpretation	
SAS data reduction	*ATSAS* v3.0
Guinier, *P*(*r*), scattering particle volume	*ATSAS* v3.0 / BioXTAS RAW
Shape/bead modeling	*DAMMIF/N* (*ATSAS* v3.0)
(*d*) Structural parameters	
**Guinier analysis**	
I(0) (cm^{-1}) / R_g (Å)	0.088 ± 0.0001 / 35.70 ± 0.09
q-range (Å^{-1})	0.007−0.036
Quality-of-fit parameter (r^2 fit)	0.84
***P*(*r*) analysis**	
I(0) (cm^{-1})	0.086 ± 0.0008
R_g (Å) / D_{max} (Å)	34.79 ± 0.03 / 90.9
q-range (Å^{-1})	0.0092−0.2214
Quality-of-fit parameter (χ^2)	0.72
M (Da) from *I*(0) (ratio to expected value)	143 866 (0.98)
Volume (V_P / V_C)	264 297 / 744
(*e*) Shape modeling results	
q-range for fitting	0.0−0.2214
Symmetry/anisotropy assumptions	*P*222 / unknown
χ^2 value/range	0.957−0.976
Model resolution (Å)	28.3

[1] ProtParam, Expasy web server at https://web.expasy.org/cgi-bin/protparam/protparam. *ox*, all Cys oxidized to cystines. *red*, all Cys taken as reduced (free thiol form).

[2] Theoretical molecular mass calculated from the primary sequence (*p.s.*).
